# Investigation of the immunological effects of escitalopram oxalate in the breast cancer co-culture model

**DOI:** 10.2478/abm-2024-0019

**Published:** 2024-06-28

**Authors:** Nalan Biriz, Zerrin Canturk

**Affiliations:** Department of Pharmaceutical Microbiology, Institute of Health Sciences, Anadolu University, Eskisehir 26470, Turkey; Department of Pharmaceutical Microbiology, Faculty of Pharmacy, Anadolu University, Eskisehir 26470, Turkey

**Keywords:** co-culture, escitalopram oxalate, MCF-7, MDA-MB-231, THP-1

## Abstract

**Background:**

During breast cancer treatment, approximately half of the patients are prescribed psychotropic medication, such as selective serotonin reuptake inhibitors (SSRIs). Escitalopram oxalate is an SSRI used as an antidepressant.

**Objectives:**

In this study, by creating a breast cancer microenvironment with THP-1, MCF-7 and MDA-MB-231 breast cancer co-culture models were created.

**Methods:**

MCF-7, MDA-MB-231, and THP-1 cell lines to determine the concentration range of the cytotoxic effect of escitalopram oxalate MTS and MTT test were used. IC_50_ values were determined by the xCELLigence real-time cell analysis (RTCA) system. Apoptotic activities and cytokine levels were determined by flow cytometry.

**Results:**

In the xCELLigence real-time analysis made according to the results, the IC_50_ value of escitalopram oxalate was measured as 13.7 μM for MCF-7 and 10.9 μM for MDA-MB-231. The IC_50_ value was measured as 54.6 μM for MCF-7 and 58.4 μM for MDA-MB-231 in xCELLigence analysis with tamoxifen. According to the MTS test results, the IC_50_ value of tamoxifen for THP-1 was 92.03 μM and the IC_50_ value for escitalopram oxalate was 95.32 μM. In the co-culture model, the immunological effects of escitalopram oxalate on MCF-7 cells were 2.8%, 11.1%, 15.6%, 10.6%, and 12.1% for interleukin (IL)-1β, IL-6, IL-8, IL-10, and TNF-α, respectively, while MDA effects on MB-231 cells, respectively, were 2.1%, 15.9%, 16.2%, 8.8%, and 11.8%.

**Conclusions:**

According to the results obtained, it was concluded that the immunological effects of escitalopram oxalate are more effective than tamoxifen and that it can be used as an adjunctive agent in breast cancer treatment.

Breast cancer is the most common type of cancer with a rate of 25.1% among women in the world [1]. In general, breast cancer is a heterogeneous type of cancer, but the formation of breast cancer subtypes is related to hormones (World Cancer Research Fund, 2017). Depending on whether the hormone receptors are positive (+) or negative (−), breast cancers show either a good prognosis or a poor prognosis [2]. Breast cancer onset and progression are linked to inflammation. The hypothesis that the immune system has a causal role in breast cancer is supported by epidemiological, preclinical, and clinical studies [3]. It has been observed that the interactions between breast cancer cells and macrophages can result in high cytokine expression, including cytokines that affect the macrophage phenotype [4].

Studies have shown that estrogen (ER)+/− cell lines differ in terms of cytokine release in breast cancer. Interleukin (IL)-6 is secreted from both ER+ and ER− cell lines. However, IL-6 secreted at a higher rate in ER-cell lines causes poor prognosis [5]. IL-8 is released from ER-cell lines at high rates, causing poor prognosis [6]. High release of IL-10 can inhibit the effect of IL-6. However, a higher rate of IL-10 causes a poor prognosis [7]. High secretion of IL-1β, the most common circulating IL-1, may cause breast cancer recurrence or prevent poor prognosis [8]. As a result of the studies, it has been observed that TNF-α, which increases tumor growth and migration, exhibits anti-tumoral effects through the high local application [9]. Selective serotonin reuptake inhibitors (SSRIs) are preferred in antidepressant treatments of patients with breast cancer during the depression process. Drugs that have an SSRI mechanism include sertraline, paroxetine, citalopram, escitalopram, fluoxetine, and fluvoxamine derivatives. These drugs are used in the treatment of obsessive-compulsive disorder and bipolar disorder as well as the treatment of depression [10]. Escitalopram, also known as (S)-citalopram, is at least 2 times more active as an SSRI than racemic citalopram. In clinical findings, it is supported that escitalopram can be administered at lower doses, therefore it has a better safety profile [11].

As a result of various studies, it has been observed that the serotonin level in the tumor has a significant effect on the development of cancer [12]. Serotonin used at low doses inhibits tumor growth by reducing the blood supply to the tumor, leading to the thought that the effect of serotonin on tumor growth is concentration-dependent [12]. Escitalopram is a type of antidepressant that has an SSRI mechanism and has low side effects [13]. Serotonin is the molecule with the highest specificity and the most selective [14]. In case studies, the effects of escitalopram oxalate on white blood cells were investigated, and no significant change was observed in the results obtained [15]. Tamoxifen, on the other hand, is an existing treatment of choice for the treatment of breast cancer. Studies have shown that tamoxifen is generally well tolerated, but it can have serious side effects depending on age [16]. Therefore, in this study, the effect of escitalopram oxalate on various types of breast cancer was tested. Studies show that antidepressant types with SSRI mechanisms may or may not cause carcinogenic effects [10]. As a result of various studies, it has been observed that the serotonin level in the tumor has a significant effect on the development of cancer [12].

Studies have generally used a single cell line; its effects on different types of breast cancer have not been compared and no relation has been established with its effects on the immune system. In this study, a co-culture model was created using macrophages, which are important components of the tumor microenvironment, and the effect of escitalopram oxalate on the immune system was investigated. The effects of many antidepressants on cancer cells have been investigated, but in this study, 2 different types of breast cancer were used instead of using a single cell line, unlike previous studies. This allowed different types of breast cancer to be compared with each other. We wanted to use the same positive control in 2 different cell lines to ensure parallelism in the experiments. MDA-MB-231 is a TNBC-type cancer cell and is treated with a taxane-derived agent. The effect mechanism of Taxan works by stopping the cell cycle in the G2 and M phases [17]. Similarly, tamoxifen stops the cell cycle in the G1 phase and also affects cancer cells thanks to its programmed cell death effect [18]. Therefore, we preferred tamoxifen in both cell lines. Also, the connection of escitalopram oxalate with the immune system was investigated with the breast cancer co-culture model. For this purpose, the THP-1 cell line was used to create the MCF-7 and MDA-MB-231 cell lines and the tumor microenvironment as breast cancer cell lines and important results were obtained.

## Materials and methods

### Chemicals and devices

- Escitalopram Oxalate (MA = 414.433 g/mol, Germany)
- Tamoxifen (MA = 371.52 g/mol, 50MG-Sigma, Germany)
- Lipopolysaccharide E. coli 0111:B4 (L4391 1MG-Sigma, Germany)
- Penicillin/Streptomycin (Biological Industries, Israel)
- Pyromycin (Invivogen, USA)
- B-Mercaptoethanol (AppliChem, Germany)
- Trypan blue (Roche, Germany)
- Trypsin-EDTA 10X (PAN-Biotech, Germany)
- MTT (Sigma-Aldrich, Germany)
- MTS (CellTiter 96® AQueous One Solution Cell Proliferation Assay, USA)
- Phosphate Buffer Salina (PBS) (Invitrogen, USA)
- RPMI-1640 medium (1X) (HyClone, Thermo Scientific, USA)
- DMEM medium (1X) (HyClone, Thermo Scientific)
- Fetal Bovine Serum (FBS) (Sigma-Aldrich)
- Dimethyl sulfoxide (DMSO) (Roth, Germany)
- Apoptosis Test Kit (Serva, Germany)
- Fix&Perm Cell Permeability Kit (Life Technology, USA)
- Interleukin 1-βB, Interleukin-6, Interleukin-8, Interleukin-10 (Biolegend, USA)
- TNF-α (Beckman Coulter, USA)
- xCELLigence E-plate 16 (Roche)
- Cedex XS (Innovatis, Almanya)
- xCELLigence Real-Time Cell Analysis (RTCA) DP System (Roche)
- Flow cytometry device (BD Accuri C6, USA)
- Laminar Flow Cabin (Heal Force)
- Cytation 3 Cell Imaging Multi-Mode Reader (ELISA reader) (BioTek, Belgium)
- Sterile CO_2_ incubator (Thermo Scientific)

### Cell lines

RPMI-1640 medium containing 1% Pen-Strep, 0.1% Pyromycin, 10% FBS, and 0.0004%-mercaptoethanol was used to grow the THP-1 (ATCC® TIB-202™) cell line. MCF-7 (ATCC® HTB-22™) cell line was developed in 10% FBS, 1% penicillin-streptomycin, 1% amphotericin-B, 0.1% puromycin, and RPMI-1640 medium. The MDA-MB-231 (ATCC® HTB-26™) cell line was developed in 10% FBS, 1% penicillin-streptomycin, 1% amphotericin-B, 0.1% puromycin, and Dulbecco's Modified Eagle's Medium (DMEM) medium. All cells were obtained from American Type Cell Culture (ATCC).

### Cytotoxicity test (MTT test and MTS test)

MTT test was performed for MCF-7 and MDA-MB-231 cells and the MTS test was applied on THP-1 cells. After the cells were counted, 5 × 10^3^ cells were calculated in each well and 100 μL was distributed to 96-well plates. The plates were incubated for 24 h in a 5% CO_2_ incubator. For tamoxifen and escitalopram, oxalate dilution was prepared in the range of 125–3.9 μM and given to each well in a 100 μL medium. After 24 h of incubation, the media were withdrawn and discarded. MTT prepared at a ratio of 1:10 in the dark was distributed to each well as 10 μL (5 mg/mL) and incubated in an incubator with 5% CO_2_ for 4 h.

MTT dye was drawn off from MCF-7 and MDA-MB-231 cells whose incubation period had expired. Later, 100 μL of DMSO was given to each well and measured at 540 nm absorbance in an ELISA reader within half an hour. THP-1 cells treated with MTS were measured directly in ELISA at 490 nm wavelength after the incubation period [19]. Cell viability was calculated from the obtained data and plotted on GraphPad, and significant results were selected for MCF-7 and MDA-MB-231 cells and tested on the xCELLigence system.

### RTCA system (xCELLigence technology)

For the MCF-7 and MDA-MB-231 cell lines, 100 μL of clean MCF-7 medium was added to the special E-plates used for the xCELLigence instrument. The xCELLigence system was stopped to install cells on the clean media that were taught, and after counting the cells in the Cedex device, 100 μL was distributed to each well except for one well by calculating 1 × 10^4^ cells per well. Medium control was performed by adding 100 μL of clean medium to the empty well. The cells, which were kept in the sterile cabinet for 15 min, were placed in the xCELLigence system and the program continued to be run from where it left off. When the number of cells measured with a clean medium for 24 h reached the highest point, the device was stopped and the E-plates were removed. 100 μL was withdrawn from the medium and the concentrations prepared in the range of 100 μL, 125 μM–3.9 μM for tamoxifen, and 30–7.5 μM for escitalopram oxalate were given. The device was restarted and measurements were taken for 48 h [20].

### Apoptosis test (Annexin V-PI)

MCF-7 and MDA-MB-231 cells were counted and added to 6-well plates at 1 × 10^5^ cells per well. Since MCF-7 and MDA-MB-231 were cell lines adhering to the surface, they were left to incubate for 24 h.

At the end of the incubation, the IC_50_ values of the substances whose apoptosis would be measured (for MCF-7: tamoxifen IC_50_ 54.6 μM, escitalopram oxalate IC_50_ 13.7 μM; for MDA-MB-231: tamoxifen IC_50_ 58.4 μM, escitalopram oxalate IC_50_ 10.9 μM) were given. Meanwhile, 1 well was allocated as the control group. Cells were allowed to incubate in a medium for 24 h.

At the end of incubation, cells were detached and washed with PBS. After washing, PBS was removed. 10X Annexin-V binding buffer kept on ice was added at 200 μL to each Falcon. 5 μL of Annexin-V and 10 μL of propidium iodide were added to the cell suspension obtained. Cell suspensions were gently vortexed and incubated for 20 min at room temperature in the dark.

After incubation, the stained cells were washed with the help of 1X binding buffer and removed by centrifugation. Finally, 400 μL 1X binding buffer was added to the cell pellet and Eppendorf was taken. In this way, cells were stained with 2 different dyes: living cells (FITC−, PE−), early apoptotic cells (FITC+, PE−), late apoptotic cells (FITC+, PE+), and necrotic cells (FITC−, PE+) [21].

### Stimulating THP-1 cells with *E. coli* LPS

After the THP-1 cells reaching the appropriate density were counted, they were inoculated into 6-well plates at 2 × 10^5^ cells per well. While *E. coli* LPS was added to one well of suspended THP-1 cells at a rate of 1 μg/mL and the other well at a rate of 100 ng/mL, 1 well was separated as the control group and left to incubate for 4 h.

THP-1 cells were collected at the end of incubation and transferred to Falcons. Each well was washed with 1 mL of cold PBS and transferred to the Falcon. THP-1 cells centrifuged at 1,100 rpm for 5 min settled to the bottom and the supernatant was removed. 300 μL of cytofix/cytoperm was added to the THP-1 cells, which were washed 2 more times with 1 mL of cold PBS and incubated on ice for 20 min. At the end of incubation, the centrifuged cells were washed twice with 500 μL perm wash and after the last wash, the cell pellet was suspended with 50 μL perm wash and 10 μL of IL-1β was added. The cells, which were incubated for 15 min in the dark and at room temperature, were washed with 500 μL perm wash at the end of incubation and finally transferred to ependorpha by adding 350 μL perm wash. The resulting suspended cells were then analyzed by flow cytometry. The suspended cell obtained after the procedure was analyzed by flow cytometry [22].

### Co-culture model and immunology experiments

MCF-7 and MDA-MB-231 cells were counted and inoculated into a 24-well plate at 2 × 10^5^ cells. MCF-7 and MDA-MB-231 cell lines were seeded into the well to create a co-culture model.

Approximately 20 h later, the THP-1 cells that developed were inoculated into the lower chamber of the 24-well plate at 2 × 10^5^ cells per well. 100 ng/mL LPS was added to the planted THP-1 cells and allowed to incubate for 4 h. At the end of the incubation period, the wells containing MCF-7 and MDA-MB-231 cells that were planted 24 h ago on the THP-1 cell groups given LPS were placed. A co-culture model was created. Concentrations at IC_50_ values obtained for MCF-7 and MDA-MB-231 were applied to the co-culture model formed and it was left to incubate for 24 h.

At the end of incubation, the chambers were removed, and the cells were collected from each well and transferred to 15 mL Falcons. Each well was washed once with 1 mL of cold PBS and transferred back to the Falcons. Meanwhile, 100 μL trypsin-EDTA was added to the wells containing the MCF-7 and MDA-MB-231 cell lines, which were allocated as the control group, and the plate was incubated for 3 min. At the end of incubation, MCF-7 and MDA-MB-231 cells were collected from the wells washed with 1 mL PBS, and added to the appropriately labeled Falcon, and all Falcons were centrifuged for 5 min at 1,100 rpm. After centrifugation, the supernatant was removed and the supernatant was removed by washing 2 more times with 1 mL of PBS.

500 μL of PBS was added to the resulting pellet and pipetted. Since IL-8 binds to the cell surface, 100 μL was separated from each Falcon and 400 μL of cytofix was added to each remaining Falcon. Meanwhile, 10 μL of IL-1β, IL-6, IL-8, IL-10, and TNF-α dyes were added to the Eppendorfs by appropriate labeling. 100 μL of cytofix-added cell suspension was distributed into the Eppendorfs to which IL-1β, IL-6, IL-10, and TNF-α were added. 100 μL of the cell suspension without cytofix was added to Eppendorf with IL-8 added. The resulting suspensions were incubated for 15 min in the dark and at room temperature.

At the end of the incubation period, 1 mL of PBS was added to each Eppendorf and centrifuged at 300–350×*g* for 5 min. After centrifugation, the supernatant was carefully removed and 200 μL of PBS was added to each Eppendorf and analyzed by flow cytometry [23].

### Statistical analysis of cytotoxicity methods

Statistical evaluations and plotting of graphs were made using the GraphPad Prizm 6.0 analysis program. During the analyses, the % inhibition graphs of the cell groups were drawn with the maximum evaluation of the control group, and the obtained data were analyzed by applying the Tukey test post hoc with one-way ANOVA.

Statistical significance values were *P* > 0.5 no difference, *P* < 0.05 (*) difference, *P* < 0.01 (**) significant difference, *P* < 0.001 (***) significant difference, and *P* < 0.0001 (****) considered to be a very significant difference.

## Results

### Cytotoxicity results of tamoxifen and escitalopram oxalate for MCF-7, MDA-MB-231, and THP-1 cell lines

**Figure 1.**
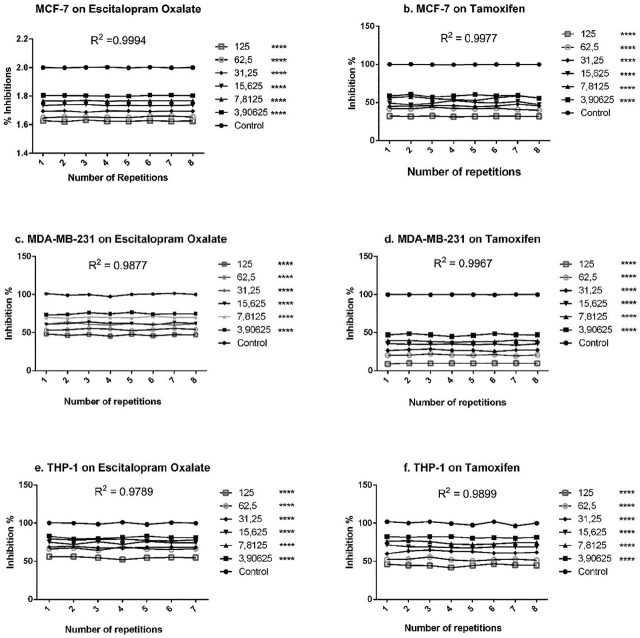
Cell viability values and statistical evaluation of escitalopram oxalate and tamoxifen (positive control) concentrations on MCF-7, MDA-MB-231, and THP-1 cell lines (****P < 0.0001, R2 is coefficient of determination).

MTT results for tamoxifen and escitalopram oxalate of MCF-7 and MDA-MB-231 cells were read at 540 nm wavelength with the aid of an ELISA reader. MTS results of THP-1 cells for tamoxifen and escitalopram oxalate were read with the aid of an ELISA reader at 420 nm wavelength. The absorbance values obtained were accepted as cell viability, and the data were created in the form of % calculation in the Microsoft Office Excel program. The control group was accepted as 100 in the results of the experiments which performed 3 replicates independently from each other, and the % values of all other concentrations were calculated according to this value. The data obtained were analyzed statistically using the GraphPad Prism 6.0 program and their graphs were created. % viable cell concentration graphs of the tamoxifen and escitalopram oxalate groups of MCF-7 and MDA-MB-231 cell lines are given in **Figure 1**. When the graph was examined, it was seen that tamoxifen gave meaningful results at higher concentrations for MCF-7 and MDA-MB-231 cell lines, while escitalopram oxalate was effective on MCF-7 and MDA-MB-231 at lower doses.

**Figure 2.**
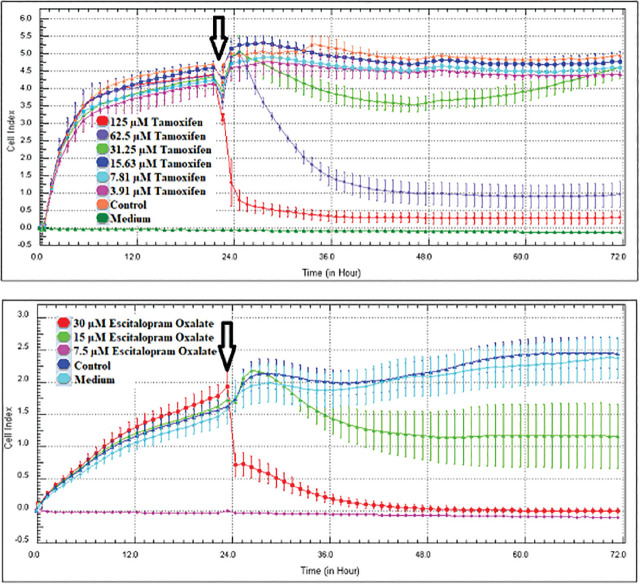

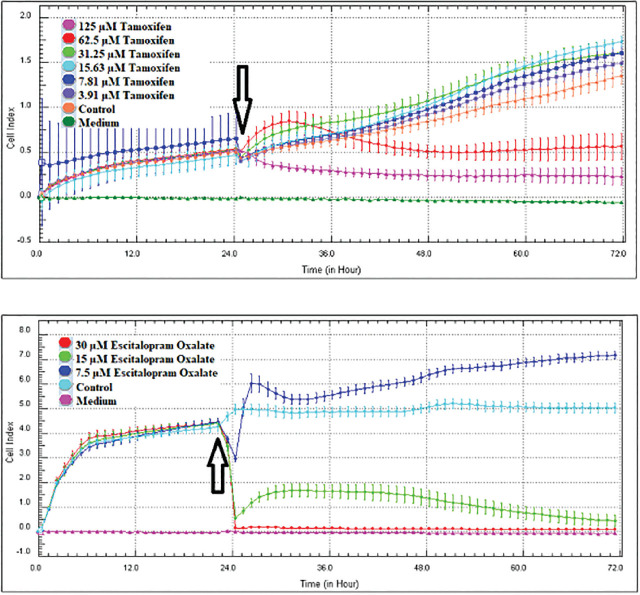
MCF-7 and MDA-MB-231 cell lines proliferation curve of 48 h concentrations of tamoxifen and escitalopram oxalate (n = 4, mean ± standard deviation). After 24 h, tamoxifen/escitalopram oxalate was given (arrowed). When the device starts to read again, the cell proliferation according to the concentrations is displayed on the graph hourly.

As a result of the screenings performed with the MTT cytotoxicity test, the concentration ranges of tamoxifen (125–3.90625 μM) and escitalopram oxalate (30–7.5 μM) of MCF-7 and MDA-MB-231 cell lines were determined and applied in the RTCA DP system. The device was stopped 48 h after application of the concentrations (72 h in total) and the IC_50_ values were automatically calculated by the RTCA DP Software 1.2.1 program. IC_50_ values of tamoxifen and escitalopram oxalate obtained for MCF-7 and MDA-MB-231 cell lines are shown in **Table 1** and **Figure 2**.

**Table 1.** IC_50_ values of tamoxifen and escitalopram oxalate for MCF-7 and MDA-MB-231 cell lines

**Cell line**	**Tamoxifen (μM)**	**Escitalopram oxalate (μM)**
MCF-7	54.6	13.7
MDA-MB-231	58.4	10.9

### Evaluation of apoptotic effects of tamoxifen and escitalopram oxalate in MCF-7 and MDA-MB-231 cell lines in flow cytometry

After applying tamoxifen and escitalopram oxalate at appropriate IC_50_ concentrations to MCF-7 and MDA-MB-231 cell lines, Annexin V-PI was administered by the procedure and the results were measured using a flow cytometer (**Figure 2)**. When MCF-7 was treated with escitalopram oxalate for 24 h (IC_50_ 13.7 μM), apoptotic effects in MCF-7 cells were 1.12 times higher than in living cells treated with escitalopram oxalate compared with the control group. Necrosis cells were 1.3 times more than the group treated with escitalopram oxalate, and apoptotic cells were 3.5 times the control.

When we compare the apoptotic effects of MDA-MB-231 with escitalopram oxalate (IC_50_ 10.9 μM) for 24 h in MDA-MB-231 cells compared with the control group, living cells are 1.05 times more than the living cells treated with escitalopram oxalate. Necrosis cells were 2.8 times more than the group treated with escitalopram oxalate, while apoptotic cells were 1.4 times the control.

**Figure 3.**
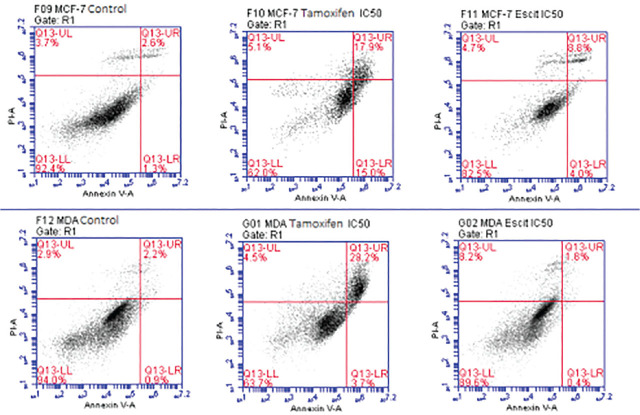
The effect of IC50 values of tamoxifen and escitalopram oxalate on MCF-7 and MDA-MB-231 cell lines by staining with Annexin-V – PI dye in flow cytometry. Q13-LL Live cell, Q13-UL Necrosis, Q13-LR Early Apoptosis, Q13-UR Late Apoptosis.

According to the results, the necrotic effects of escitalopram oxalate were similar to tamoxifen in MCF-7 cells, while it was higher in MDA-MB-231 cells than tamoxifen. Its apoptotic effects are not as effective as tamoxifen in MCF-7 and MDA-MB-231 (**Figure 3**).

### Evaluation of the immunological effects of tamoxifen and escitalopram oxalate in the created breast cancer co-culture model

IC_50_ concentrations were given to breast cancer co-culture models (MCF-7/THP-1 and MDA-MB-231/THP-1) planted and merged according to the procedure, and after 24 h using flow cytometry, IL-1β, IL-6 levels of IL-8, IL-10, and TNF-α were measured.

The immunological effects of tamoxifen and escitalopram in the MCF-7 co-culture model are shown in **Table 2**.

**Table 2.** Results for IL-1β, IL-6, IL-8, IL-10, and TNF-α in MCF-7 co-culture model (percent positive sign indicates amount secreted)

		**IL-1β**		**IL-6**		**IL-8**		**IL-10**		**IL-α**	
				
**Cell lines**	**Substances**	**% (−)**	**% (+)**	**% (−)**	**% (+)**	**% (−)**	**% (+)**	**% (−)**	**% (+)**	**% (−)**	**% (+)**
THP-1	Control	96.2	3.8	91.1	7.7	85.5	14.2	90.0	9.7	86.9	12.7
	100 ng/mL LPS	57.6	42.4	76.4	20.1	53.7	44.4	77.3	22.1	68.6	30.9
MCF-7	Control	93.8	6.2	81.2	18.5	77.3	20.2	88.6	11.0	79.8	19.9
Co-culture	Control	89.6	10.4	89.4	10.3	42.7	56.1	65.8	33.3	54.8	44.2
	Tamoxifen IC_50_	94.1	5.9	80.6	19.2	80.1	18.7	87.0	12.6	78.4	21.1
	Escitalopram oxalate IC_50_	97.2	2.8	88.7	11.1	83.8	15.6	88.9	10.6	87.7	12.1

IL, interleukin.

**Figure 4.**
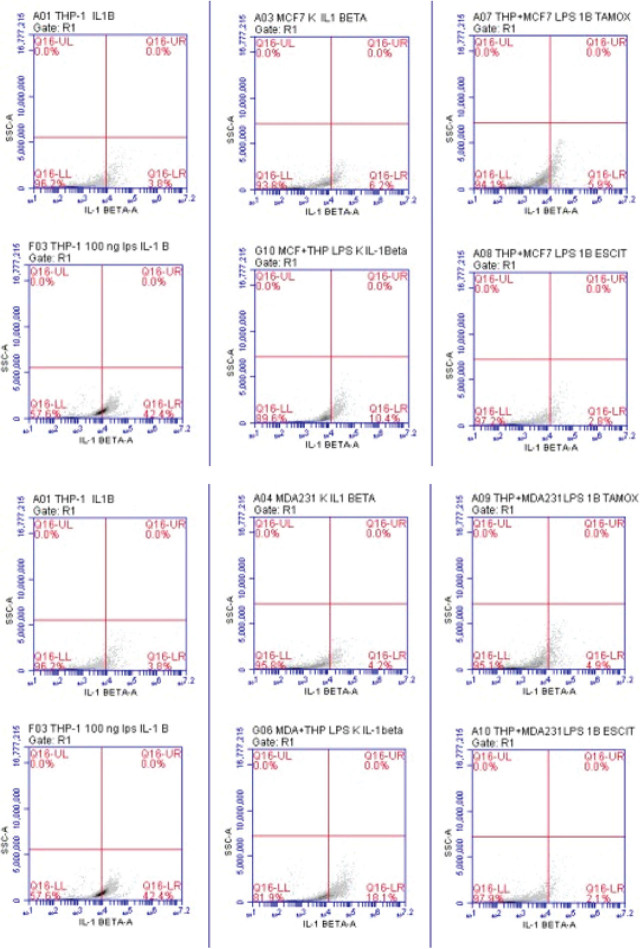

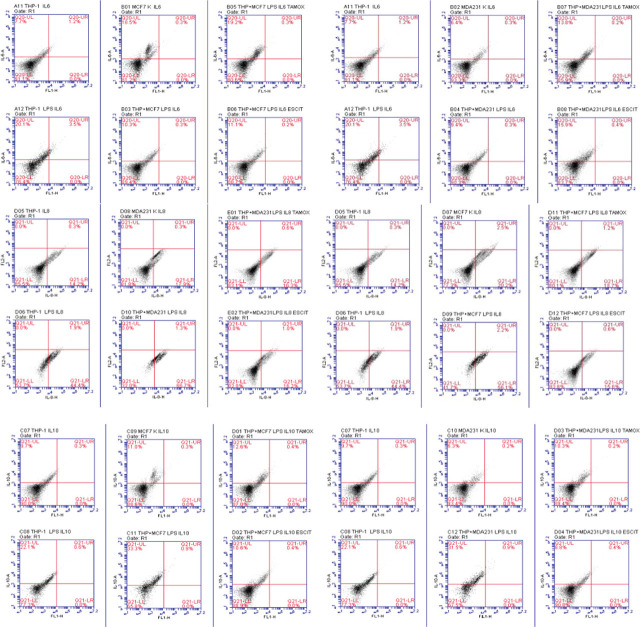
IL-1β, IL-6, IL-8, IL-10 and TNF-α levels in the MCF-7 and MDA-MB-231 co-culture model. Q16-LR amount of secreted IL-1β, Q20-IL amount of secreted IL-6, Q20-UL amount of secreted TNF-α, Q21-LR amount of secreted IL-8, and Q21-UL amount of secreted IL-10. IL, interleukin.

As a result of the application of 100 ng/mL LPS to the results of the measurements taken using flow cytometry, the level of IL-1β in the THP-1 control group increased by 11.15 times. IL-1β level in MCF-7 increased 1.67 times after the co-culture model was created. The IL-1β level decreased by 1.76 fold after treatment of the co-culture model with tamoxifen, and 3.71 fold after treatment with escitalopram oxalate (**Table 3** and **Figure 4**).

**Table 3.** Results for IL-1β, IL-6, IL-8, IL-10, and TNF-α in the MDA-MB-231 co-culture model (percent positive sign indicates amount secreted)

		**IL-1β**		**IL-6**		**IL-8**		**IL-10**		**IL-α**	
				
**Cell lines**	**Substances**	**% (−)**	**% (+)**	**% (−)**	**% (+)**	**% (−)**	**% (+)**	**% (−)**	**% (+)**	**% (−)**	**% (+)**
THP-1	Control	96.2	3.8	91.1	7.7	85.5	14.2	90.0	9.7	86.9	12.7
	100 ng/mL LPS	57.6	42.4	76.4	20.1	53.7	44.4	77.3	22.1	68.6	30.9
MDA-MB-231	Control	95.8	4.2	93.3	6.4	81.8	17.9	93.4	6.3	92.4	7.4
Co-culture	Control	81.9	18.1	90.3	9.4	32.0	66.7	67.5	31.5	49.3	48.6
	Tamoxifen IC_50_	95.1	4.9	85.9	13.8	83.1	16.3	89.4	10.3	75.5	24.0
	Escitalopram oxalate IC_50_	97.9	2.1	83.7	15.9	82.8	16.2	90.8	8.8	87.9	11.8

IL, interleukin.

In the measurements taken from the control group of THP-1 cells, the IL-6 level increased 2.61 times as a result of the application of 100 ng/mL LPS. IL-6 level in MCF-7 decreased 1.79 times after the co-culture model was created. In the co-culture model treated with tamoxifen, the IL-6 level increased 1.86-fold and 1.07-fold after treatment with escitalopram oxalate. According to the results of the measurements taken using flow cytometry, in the measurements taken from the control group of THP-1 cells, the IL-8 level increased 3.12 times by applying 100 ng/mL LPS. After the co-culture model was created, the level of IL-8 in MCF-7 increased 2.77 times. IL-8 level decreased 3-fold in the co-culture model treated with tamoxifen, and 3.59-fold in the co-culture model treated with escitalopram oxalate (**Table 2** and **Figure 4**).

In the THP-1 control group, the IL-10 level increased 2.27 times as a result of the administration of 100 ng/mL LPS. The MCF-7 control group increased 3.02 times in the measurements taken after the co-culture model was created. As a result of tamoxifen treatment, the IL-10 level in the control group decreased 2.64 times and 3.14 times after treatment with escitalopram oxalate (**Table 2** and **Figure 4**).

According to the results of the measurements taken using the flow cytometry, the TNF-α level in the measurements taken from the control group of THP-1 cells increased 2.43 times as a result of the application of 100 ng/mL LPS. The MCF-7 control group increased 2.22 times in the measurements taken after the co-culture model was created. It decreased 2.09-fold after treatment with tamoxifen and 3.65-fold after treatment with escitalopram oxalate (**Table 2** and **Figure 4**).

As a result of the measurements taken using the flow cytometry, the IL-1β level in the THP-1 control group increased 11.15 times as a result of the application of 100 ng/mL LPS. After the co-culture model was established, the IL-1β level in MDA-MB-231 cells increased 4.3-fold. This level decreased 3.7 times after treatment with tamoxifen and 8.61 times after treatment with escitalopram oxalate (**Table 3** and **Figure 4**).

In the measurements taken from the control group of THP-1 cells, the IL-6 level increased 2.61 times as a result of the application of 100 ng/mL LPS. After the co-culture model was established, IL-6 levels in MDA-MB-231 cells decreased 1.46 times. In the co-culture model treated with tamoxifen, the IL-6 level increased by 1.46 fold and 1.7 fold after treatment with escitalopram (**Table 3** and **Figure 4**).

According to the results of the measurements taken using flow cytometry, the IL-8 level in the measurements taken from the control group of THP-1 cells increased 3.12 times as a result of the application of 100 ng/mL LPS. After the co-culture model was established with the MDA-MB-231 cell, the IL-8 level increased 3.72-fold. In the co-culture model treated with tamoxifen, the level of IL-8 decreased 4.09-fold and 4.11-fold after treatment with escitalopram oxalate (**Table 3** and **Figure 4**).

In the THP-1 control group, the IL-10 level increased 2.27 times as a result of the administration of 100 ng/mL LPS. After the co-culture model was established, the IL-10 level in MDA-MB-231 cells increased 5-fold. In the co-culture model treated with tamoxifen, this level was decreased 3.05-fold and 3.57-fold after treatment with escitalopram oxalate. According to the results of the measurements taken using the flow cytometry, the TNF-α level in the measurements taken from the control group of THP-1 cells, and the TNF-α level increased 2.43 times as a result of the application of 100 ng/mL LPS. In the co-culture model created, the TNF-α level in MDA-MB-231 cells increased 6.56 times. This level decreased by 2.03 times after treatment with tamoxifen and 4.11 times after treatment with escitalopram oxalate (**Table 3** and **Figure 4**).

When comparing escitalopram oxalate and tamoxifen in the MCF-7 co-culture model, it appears that escitalopram oxalate inhibits the level of cytokine secretion better. Also, the level of IL-6 continued but was less than tamoxifen. Similarly, in the MDA-MB-231 co-culture model, escitalopram inhibited oxalate cytokine levels better than tamoxifen, and likewise, the increase in IL-6 levels continued.

Also, when we compare the MCF-7 and MDA-MB-231 cell lines among themselves, it is seen that escitalopram oxalate is more effective in both cell lines, while the decrease in cytokine levels in the MDA-MB-231 cell line is much more striking.

## Discussion

Studies have investigated the effects of many types of antidepressants against different types of cancer. We conducted this study within the scope of the thesis, and we investigated the cytotoxic, apoptotic, and anti-inflammatory effects of escitalopram oxalate in the SSRI group in the breast cancer co-culture model.

In the studies of Xu et al. [24], tamoxifen was observed to inhibit the growth of MCF-7 cells at higher concentrations, a while 10^−7^ M concentration did not make a significant difference in the RTCA DP studies with MCF-7. In his RTCA DP study, which he conducted in his doctoral dissertation, he saw that the IC_50_ value of MCF-7 cells for tamoxifen was 22 μM [25]. Cansaran-Duman et al. [26] found the IC_50_ value of tamoxifen as 1.26E-05M in the RTCA DP they made using the MDA-MB-231 cell line. xCELLigence RTCA system is a very reliable, hourly data generation and expensive system. In the literature reviews, tamoxifen appears to be effective at low doses. In our study, the cytotoxic effect of tamoxifen on MCF-7 is more than MDA-MB-231 and it is seen that it is effective at concentrations >10^−7^ M.

Considering the studies conducted with escitalopram oxalate, although there are no studies with MCF-7 in the literature, in a study conducted by Arunasree [27] with MDA-MB-231, the IC_50_ value of escitalopram was found to be 392.24 μM. This result was obtained as a result of the MTT cytotoxicity test, and the test results were evaluated not instantaneously, but at the end of 24 h. Since the results we have obtained in the experiments we have done with RTCA DP are taken with instant evaluations and are automatically calculated values, they give more precise results. No results of escitalopram oxalate obtained by RTCA DP in breast cancer cell lines have been found in the literature.

In the literature review by Mutee et al. [28], the apoptotic effect in MCF-7 cells treated with tamoxifen was viable, necrosis/late apoptosis, and early apoptosis effects at 52.2%, 5.5%, and 41.3%, respectively. In the study by Yaacob et al. [29], 27% apoptosis was observed in MCF-7 cells treated with 2.5 μM tamoxifen for 24 h and 40% at the end of 48 h. In MDA-MB-231 cells, 2.5 μM tamoxifen was found to induce apoptosis by approximately 11% and 5 μM tamoxifen by up to 25. In the literature reviews, it is supported by studies that tamoxifen induces the apoptotic effect depending on time and concentration. Also, studies have shown that the apoptotic effects of tamoxifen are high. In our study, the amount of tamoxifen (IC_50_) applied to MCF-7 and MDA-MB-231 cells showed an apoptotic effect, but its necrotic effect was not high.

Chanput et al. [30], who stimulated THP-1 cells with LPS in their literature review, also looked at the levels of IL-1β, IL-6, IL-8, IL-10, and TNF-α, and as a result found that the level of IL-8 was strikingly high. Also, they noted that cytokines other than TNF-α and IL-10 continued to accumulate during the incubation period. Based on these data, IL-1β was the most secreted cytokine compared with the control group, and the secretion of all other cytokines increased after LPS stimulation. In our study, an increase was observed in cytokine levels following the literature.

Ortiz-Montero et al. [31], who treated MCF-7 cells with IL-6 or IL-8 in SCM, found that IL-1β, IL-6, and IL-8 were the supernatants of both developed and undeveloped MCF-7 cells in SCM. IL-8 measured the amounts of IL-10 and TNF-α and found that IL-6 and IL-8 were quite rich. In the studies conducted by Kozlowski et al. [7], they found that high levels of IL-6, IL-8, and IL-10 were associated with the clinical stage of breast cancer. Also, they concluded that IL-10 both produced an immune cell type response and could lead to the progression of malignancy by producing IL-6 and IL-8. In the study of Chen et al. [32], the highest level of IL-6 was measured in MCF-7 cells, while the highest level of IL-8 was measured in MDA-MB-231.

In our co-culture study, tamoxifen and escitalopram oxalate decreased IL-1β secretion in both MCF-7 and MDA-MB-231 cells, but escitalopram oxalate decreased IL-1β levels much more than tamoxifen.

IL-6 makes cancer cells more aggressive. There are some results in the studies that support this. One of these is that MCF-7 cells display more aggressive properties in the aging-conditioned environment and the level of IL-6 secreted by MCF-7 cells in this environment increases [33]. In our experiments, after the treatment of cells with tamoxifen and escitalopram oxalate, there was no decrease in the level of IL-6 in the environment; on the contrary, tamoxifen caused a higher rate of IL-6 secretion than escitalopram oxalate.

IL-8, which plays a role in breast cancers that is mostly determined by ER and HER2 expression, is highly expressed, and ER+/− increases metastasis in both breast cancers. On the other hand, in breast cancers that express HER2 at low levels such as MCF-7, HER2 re-expression may cause higher IL-8 expression [33]. In our co-culture study, both tamoxifen and escitalopram oxalate reduced IL-8 levels. Although the effect of escitalopram oxalate appears to be more effective than tamoxifen, the difference is not much.

IL-10, which is responsible for controlling cellular immunity, may play a role in suppressing immunity in breast cancer, as well as creating an antitumor response. In the literature, it is stated that IL-10 is more expressed in ER− tumors compared with ER+ tumors and IL-10 level is less in PR+ patients [33]. According to the experimental results, tamoxifen and escitalopram oxalate decreased IL-10 levels in both cells.

TNF-α, which is highly expressed in breast tumors, is released by tumor cells in response to LPS [34]. Inhibition of TNF-α is protective against chemically induced breast tumors. According to the literature, TNF-α supports the growth of the tumor, but there are also data showing that it differs between breast cancer cells [9]. In our co-culture study, escitalopram oxalate decreased TNF-α levels more than tamoxifen. When we look at all this literature information and results, our experiment results are supported by the literature. However, the fact that IL-6 levels are still high after substance administration does not comply with the literature.

There is not enough data in the literature regarding the effects of escitalopram oxalate on MCF-7 and MDA-MB-231. Therefore, it has not been easy to compare measurements (such as cytokine levels) that have been studied in detail. However, considering the obtained results, it is seen that the effects of escitalopram oxalate on breast cancer cells should be investigated in more detail.

Also, the immune system responses (IL-1β, IL-6, IL-8, IL-10, and TNF-α) that we examined with the co-culture model gave better results in escitalopram oxalate. However, cytokine levels in MCF-7 cells exposed to escitalopram oxalate were lower than those of MDA-MB-231. The reason for this may be the inflammatory response caused by necrosis in MDA-MB-231 cells, as well as the immune response created by escitalopram oxalate in the environment.

Therefore, we believe that this study will contribute to the effects of escitalopram oxalate, which has an SSRI mechanism, on breast cancer.
